# In Vitro Evaluation of Experimental Self-Adhesive Orthodontic Composites Used to Bond Ceramic Brackets

**DOI:** 10.3390/ma12030419

**Published:** 2019-01-30

**Authors:** Ana Carolina Costa, Vicente Sabóia, Felipe Marçal, Nara Sena, Diego De Paula, Thyciana Ribeiro, Victor Feitosa

**Affiliations:** 1 Department of Restorative Dentistry, Federal University of Ceará, Fortaleza 60430-355, Brazil; carolinafigueired@gmail.com (A.C.C.); vpsaboia@yahoo.com (V.S.); felipef.marcal@gmail.com (F.M.); narajuliana@gmail.com (N.S.); thyciana_odonto@yahoo.com.br (T.R.); 2 Research Division, Paulo Picanço School of Dentistry, Fortaleza 60325-218, Brazil; dmartins1987@hotmail.com

**Keywords:** orthodontic brackets, composite resins, dental materials

## Abstract

The aim of this study was to evaluate the degree of conversion (DC), flexural strength (FS), and shear bond strength (SBS) of ceramic brackets bonded to enamel with experimental self-adhesive orthodontic composites. Functional monomers 10-methacryloyloxy-decyl-dihydrogen-phosphate (MDP) and glycerol-dimethacrylate-phosphate (GDMA-P) were used in experimental composites. They were compared to the same composite without an acidic monomer (negative control) and with enamel acid-etching prior to adhesive application (positive control). DC was evaluated by Raman micro-spectroscopy. Flexural three-point bending testing was performed in a universal testing machine. Ceramic brackets were bonded to bovine enamel and SBS was evaluated before and after 2000 thermal-cycles. Fracture patterns were surveyed with manual removal with specific pliers and analyzed by SEM. Statistics was performed using ANOVA and Tukey’s test (*p* < 0.05). DC of the control composite was significantly higher (*p* < 0.001) than that of GDMA-P and MDP. FS showed no significant difference between composites (*p* = 0.451). Regarding adhesion, the positive control (8.47 ± 0.88 MPa) and MDP (7.07 ± 2.69 MPa) obtained higher overall results. The predominant fracture pattern of the positive control and MDP was mixed while it was adhesive for further groups. The MDP-containing orthodontic composite attained similar adhesion to a conventional three-step orthodontic bonding system, with a similar fracture pattern and mechanical properties. Nevertheless, the presence of acidic functional monomers reduced the DC.

## 1. Introduction

The traditional gold standard etching protocol for orthodontic bracket bonding presents enamel acid etching with 37% ortho-phosphoric acid and the application of adhesive for posterior application of an orthodontic composite [1,2]. The introduction of acid etching to the bonding procedure of fixed appliances has been one of the most significant changes in orthodontics [3,4]. It creates micro-retentions on the enamel surface which aid the feasible process of fixing the bracket. However, it may make the bracket removal process more laborious, thereby causing irreversible damage to the enamel [3,5].

Ceramic brackets are an essential part of current orthodontic practice due to their aesthetic advantage [6,7]. However, their high brittleness may disrupt their removal as a monoblock, leading to more fractures of the enamel [8,9]. A suitable alternative to reduce this problem would be the usage of an orthodontic composite with lower bond strength in order to ease bracket detachment, attaining less damage to the tooth structure [1,10]. Therefore, for ceramic brackets, the orthodontic composite might be able to accomplish a balance between enough bond strength to maintain the adhesion to enamel throughout the treatment, and lower final bond strength in order to attain minimal enamel fractures [11,12]. Indeed, the development of a self-adhesive orthodontic composite may simplify the bracket bonding process, reducing clinical chair time and likely providing less enamel damage.

The effects of commercial self-adhering flowable composites [13] as well as self-adhesive luting resin cements [10] on the shear bond strength of metallic orthodontic brackets have been well documented. Nevertheless, there is a lack of studies on the mechanical properties and adhesion of these materials associated with ceramic brackets, especially with experimental model composites comprising different acidic monomers. Furthermore, the role of acidic functional monomers on the physicochemical properties of composites and enamel fracture patterns after manual removal requires deeper investigation.

Thus, the aim of the present study was to evaluate the degree of conversion, flexural strength, and enamel shear bond strength before and after the thermocycling of experimental self-adhesive orthodontic composites associated with ceramic brackets, and the fracture pattern after the simulation of clinical bracket removal. The hypotheses tested are: (1) experimental orthodontic composites exhibit physicochemical properties similar to conventional control composites, (2) experimental orthodontic composites achieve similar enamel bond strength to a conventional three-step technique with a control composite, and (3) experimental composites promote less damage to enamel after the manual removal of brackets than a control composite.

## 2. Materials and Methods 

### 2.1. Formulation of Experimental Composites

**Figure 1:**
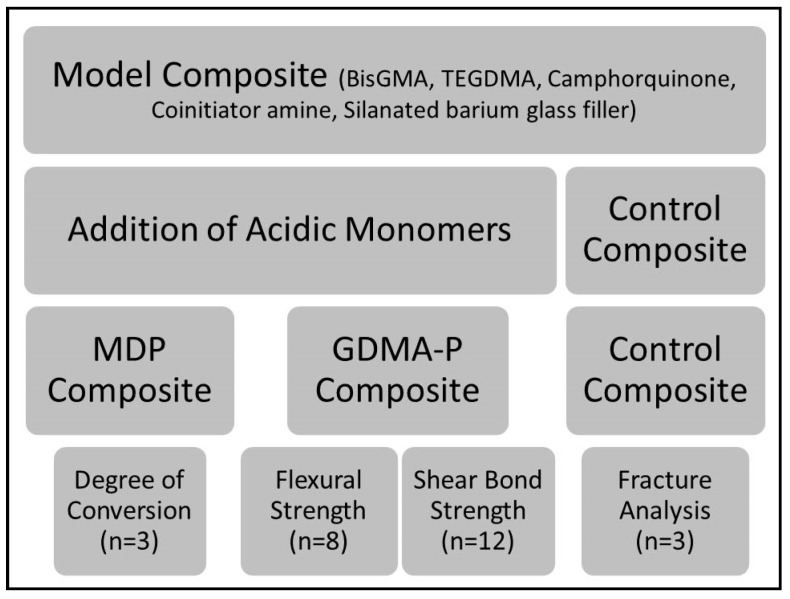
Flow chart summarizing main information about experimental protocol, materials, and testing steps. Legend: BisGMA—bisphenol-A-glycidil dimethacrylate, TEGDMA—triethylene glycol dimethacrylate, MDP—10-methacryloyloxy-decyl-dihydrogen-phosphate, and GDMA-P—glycerol-dimethacrylate-phosphate.

The control composite (without an acidic monomer) was prepared using a similar composition to Transbond XT (3M, St. Paul, MN, USA), consisting of 60 wt % silanated barium glass fillers (0.7 µm mean size, donated by Esstech Inc., Essington, PA, USA), 10 wt % triethylene glycol dimethacrylate (TEGDMA), 28 wt % bisphenol-A-glycidil dimethacrylate (BisGMA), 1 wt % camphorquinone (CQ, photoinitiator) and 1 wt % ethyl-dimethylamine benzoate (EDAB, co-initiator). In the experimental composites, 10 wt % of the acidic functional monomers glycerol-dimethacrylate-phosphate (GDMA-P) or 10-methacryloyloxy-decyl dihydrogen phosphate (MDP), both donated by Yller Biomaterials (Pelotas, Brazil), were added, replacing TEGDMA. The experimental adhesive (used only with the control composite) was prepared with 40 wt % TEGDMA, 58 wt % BisGMA, 1 wt % CQ, and 1 wt % EDAB. The negative control was the control composite which was free of acidic monomers and was used without adhesive. All blends were hand-mixed until consistency was homogeneous and initiators had dissolved. Fillers were added under magnetic stirring and the final mixture was ultrasonicated for 3 min to remove air bubbles. Experimental protocols are summarized in Figure 1.

### 2.2. Degree of Conversion

The degree of conversion of experimental and control composites was evaluated using a previous described protocol [14]. Briefly, three milligrams of each composite were analyzed using Raman Spectroscopy (Xplora, Horiba Jobin Yvon, Paris, France) with a HeNe laser using 3.2 mW power and 532 nm laser wavelength. Percentage conversion was surveyed by the ratio of peaks 1610/1637 cm^−1^ before and subsequent to 40 s light-activation (DB685, 1100 mW/cm^2^, Dabi Atlante, Ribeirao Preto, Brazil). The acquisition time was ten seconds with three accumulations. The analyses were performed in triplicate. 

### 2.3. Three-Point Bending Test

Bar-shaped specimens (n = 8) of experimental and control composites were created using silicone molds (Scan Putty, Yller Biomaterials, Pelotas, Brazil). The samples’ dimensions were 0.5 mm in thickness, 1 mm in width, and 8 mm in length. The experiment was performed using a previously published protocol [14] according to ISO 4049:2009. The composites were inserted into the molds, a polyester strip was placed onto the composites, and the tip of the DB-685 LED (1100 mW/cm²; Dabi Atlante, Ribeirao Preto, Brazil) was placed on the top of the sample while the curing was realized for 40 s. The tip diameter (10 mm) covered the entire specimen, allowing a single activation for each sample. The specimens were carefully removed from the molds, those with defects were discarded, and the suitable samples were stored in a 100% relative humidity environment for 24 h. Before testing, composite bars were measured using a digital caliper (Mitutoyo, Tokyo, Japan). Subsequently, the specimens were submitted to three-point bending testing in a Universal Testing Machine (DL 2000, EMIC-Instron, São José dos Pinhais, Brazil) with a 500 N load cell and 0.5 mm/min crosshead speed to obtain maximum flexural strength.

### 2.4. Shear Bond Strength (SBS)

#### 2.4.1. Selection and Preparation of Samples

Bovine incisors with intact enamel surfaces were selected, cleaned, and stored in 0.1% thymol solution at 37 °C. The roots were sectioned at the cementum-enamel junction using a cutting machine (Isomet 1000, Buehler, Lake Bluff, IL, USA) with diamond blade at low speed, and water cooled. The crown specimens were embedded in transparent acrylic resin (JET, Ribeirão Preto, Brazil) and were polished with pumice and distilled water. 

The ceramic brackets (Morelli, Sorocaba, Brazil) were bonded to the buccal enamel surface of the specimens (n = 12) according to one of four strategies based on the orthodontic composite (positive Control, negative Control, GDMA-P, or MDP) which were further divided into with or without thermocycling (2000 cycles between 5 ± 2 °C and 55 ± 2 °C for 15 s each, TC45, Peter Huber Kältemaschinenbau AG, Frankfurt, Germany). Details of each group are described in Table 1. In the groups negative Control, MDP and GDMA-P, the enamel surface was dried with a 10 s air-jet before composite application. In the positive control group, the enamel surface was etched with 37% phosphoric acid gel (Condac 37, FGM, Joinville, Brazil) for 30 s, washed with distilled water for 60 s, and vigorously air-dried; following this, the experimental adhesive was applied and light-cured using the DB-685 LED unit (1100 mW/cm²; Dabi Atlante, Ribeirao Preto, Brazil) for 40 s, after which the control composite was applied.

#### 2.4.2. Shear Bond Strength Test

The samples were subjected to shear bond strength testing with knife-type devices adapted in the universal test machine (DL 2000, EMIC-Instron, São José dos Pinhais, Brazil) with 1 mm/min crosshead speed using a 500 N load cell until bracket debonding, according to ISO 11405:1994. After failure, the samples were analyzed under optical microscopy (Leica, Heidelberg, Germany) with 60× magnification to verify the predominant fracture. Fractures from the shear bond strength test were classified as: adhesive (i.e., when it occurred only between the composite and enamel interface), cohesive in bracket (i.e., when there was fracture of the bracket), cohesive in composite (i.e., when a complete cohesive failure occurred entirely in the composite), and mixed (i.e., with partial failure occurring at the interface and partial cohesive in the composite). 

### 2.5. Clinical Fracture Pattern

Three representative specimens from each group were analyzed to evaluate the clinical simulation of bracket removal with specific orthodontic pliers performed by the same operator with previous experience and training. These fractures were analyzed by field-emission-gun scanning electron microscopy (Quanta FEG 450, FEI, Amsterdam, The Netherlands) at 20.00 kV with 100×, 200×, and 2000× magnifications. The fracture patterns had the same classification as has been previously mentioned for SBS analysis. 

### 2.6. Statistical Analysis

A two-way ANOVA test was used to determine the statistically significant differences in SBS surveying. The data of the flexural modulus and degree of conversion were statistically analyzed using one-way ANOVA and Tukey’s test. Such tests were performed after all data had passed a Shapiro-Wilk normality test (*p* > 0.05). Analyses were performed using SigmaStat 3.5 statistical software (Systat, San Jose, CA, USA). The level of significance was considered to be 5%. 

## 3. Results

### 3.1. Degree of Conversion 

**Figure 2:**
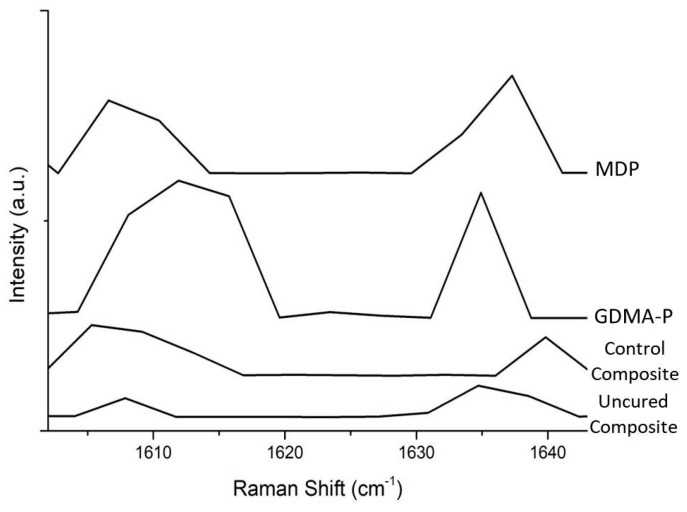
Representative Raman graphs showing the intensities of C=C vibrations of different materials.

The outcomes (means and standard deviations) of degree of conversion are presented in Table 2. The control composite obtained significantly higher conversion than the composites with acidic functional monomers (*p* < 0.05). Representative Raman graphs for all polymerized composites and the uncured composite are presented in Figure 2. 

### 3.2. Three-Point Bending Test

**Figure 3:**
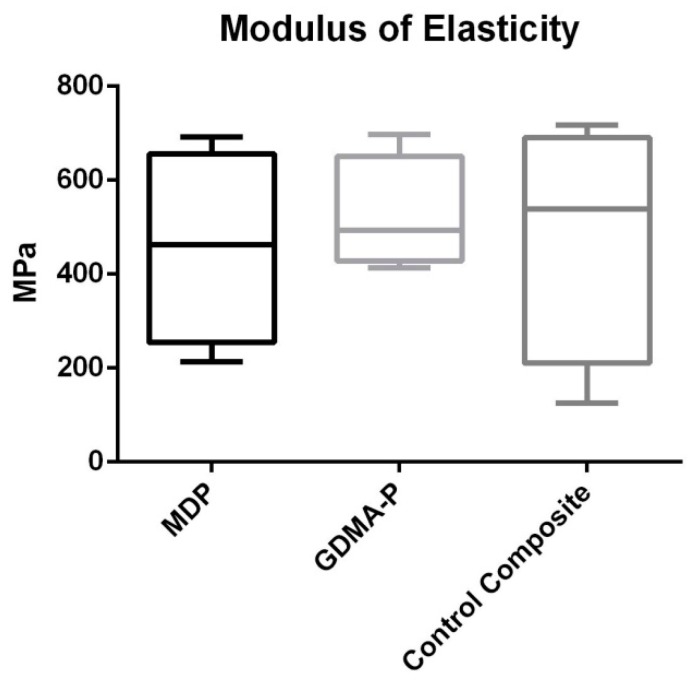
Results of a three-point bending test showing the flexural strength (MPa) of each composite. No statistical difference was observed (*p* > 0.05).

Results of flexural strength are depicted in Figure 3. No statistically significant difference was found among the composites (*p* = 0.562).

### 3.3. Shear Bond Strength Test

In shear bond strength testing, the positive control and MDP-containing composite achieved the highest outcomes before thermocycling (*p* = 0.01). After thermocycling, the bond strengths of all strategies were significantly reduced and there was no statistical difference among the composites (*p* = 0.751). The predominant fracture pattern of the positive control and the MDP composite was that of mixed fracture, whereas other groups attained mainly adhesive failures. The means and standard deviations of SBS are presented in Table 3. 

### 3.4. Clinical Fracture Pattern

**Figure 4:**
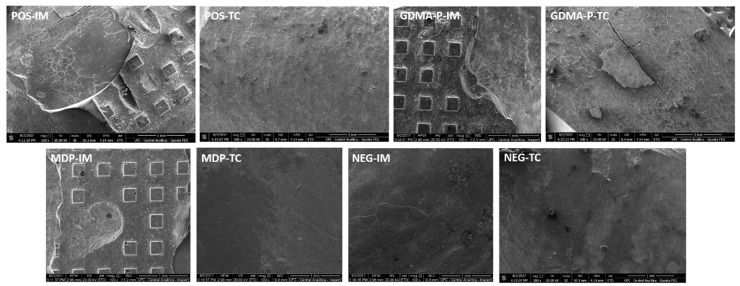
Representative scanning electron micrographs of specimens of bovine enamel after manual removal (clinical simulation) of ceramic brackets bonded with control or experimental composites. Images POS-IM, GDMA-P-IM, GDMA-P-TC, and MDP-IM depict mixed fractures, while images MDP-TC, NEG-TC, and NEG-IM show adhesive failures. Legend: POS—positive control, NEG—negative control, MDP—composite containing MDP, GDMA-P—composite containing GDMA-P, IM—immediate (before thermocycling), and TC—thermocycling (specimens after undergoing thermocycling challenge).

Representative micrographs of specimens from bracket manual removal analysis (clinical simulation) are shown in Figure 4. The MDP composite obtained mixed fractures in the immediate test and mixed/adhesive fractures after thermocycling. The composite incorporated with GDMA-P obtained mixed and adhesive fractures immediately and a premature adhesive failure after thermocycling. The negative control obtained only adhesive premature failures before and after thermocycling. The positive control attained mixed and adhesive fractures immediately and after thermocycling. This section may be divided by subheadings. It should provide a concise and precise description of the experimental results and their interpretation, as well as the experimental conclusions that can be drawn.

## 4. Discussion

The results of the present study support the rejection of the study hypotheses. The degree of conversion of the control composite was significantly higher than those of the experimental composites, although there was no statistically significant difference among flexural strength values of the composites. Regarding the bond strength, only the experimental composite with MDP presented immediate shear bond strength similar to that of the positive control (with enamel acid-etching and adhesive application). 

In this investigation, the addition of both acidic functional monomers significantly dropped the degree of conversion in comparison with the control composite. Indeed, this is thanks to the fact that these monomers are more hydrophilic and present phosphate functionalities, inhibiting the polymerization initiation by the acid-base reaction of the acidic monomer with the amine co-initiator [15,16]. Indeed, lower conversion in experimental composites may yield higher monomer elution and release from enamel areas, which could potentially provoke cytotoxicity to gingival fibroblasts, inducing inflammation. This might be a suitable topic for future investigation.

When comparing the experimental groups, it may be observed that the GDMA-P-containing composite demonstrated a higher degree of conversion than the composite with MDP. A suitable explanation for this relies on the molecular structure of acid functional monomers. GDMA-P is a di-methacrylate, able to polymerize about twice as much as MDP, which is a mono-methacrylate [17]. In addition, MDP has a larger spacer carbon chain, which further hinders the polymerization process due to its lower molecular mobility [17]. Nevertheless, it is worth mentioning that the present outcomes may be different from those of in situ degree of conversion, where the composite is light-cured after luting the orthodontic bracket. Indeed, in the clinical scenario, phosphate functionalities of acidic monomers in the composite would react with the dissolved hydroxyapatite, thereby leading to minor acid-base reaction with the amine co-initiator, promoting higher polymerization than in in vitro tests [15]. 

In spite of reducing the degree of conversion, MDP undertakes intermolecular interactions by Van der Waals forces, increasing internal cross-links and improving mechanical properties [17,18]. The mechanical strength of orthodontic composites is essential in order to aid dissipation of forces during the mechanics of orthodontic treatment. In the present investigation, the flexural strength outcomes (Figure 2) showed that the acidic functional monomers did not interfere with flexural strength. A feasible explanation for this is related to the intermolecular cross-links promoted by MDP and the higher di-methacrylate cross-linking obtained by adding GDMA-P, which is a dimethacrylate [19,20]. Both could have compensated for the lower degree of conversion of experimental composites. 

In shear bond strength surveying, the two acidic functional monomers showed adequate performance, as observed in the pilot study when further acidic monomers were evaluated (HEMA-phtalate and HEMA-phosphate). They were not included in the current research thanks to the very low initial shear bonded strength presented which was below the clinically acceptable minimum of 5 MPa [21]. Furthermore, both acidic monomers replaced TEGDMA due to similar molecular weights, thereby maintaining the balance of the resin blend.

In the present study, the addition of MDP and GDMA-P accomplished noteworthy increases in bond strength as compared to the control composite (negative control). However, only the MDP-containing orthodontic composite attained similar bond strength to the positive control before and after thermocycling, thereby demonstrating its clinical viability to be used. Nowadays, MDP is considered the gold-standard acidic functional monomer in the formulation of self-etching adhesives as it provides adequate and stable bond strength [17,22,23]. This is due to its large hydrophobic spacer chain with ten carbons that separate well the polymerizable methacrylate from the dihydrogen phosphate functionality, reacting chemically with the hydroxyapatite of the enamel through a strong chemical interaction [17,24,25]. Indeed, this might have contributed to the relatively high initial bond strength of the MDP-containing self-adhesive orthodontic composite.

When considering the bond strength after thermocycling, all values reduce strikingly. This phenomenon occurs due to degradation of the bonding interface as well as degradation of the composite itself. The main mechanism proposed for such degradation is the water breakdown of ester bonds in methacrylates, which is a link prone to hydrolysis over time. Although it is a challenge for researchers, thermocycling is a widely used method for aging samples in vitro and might have simulated well the period of orthodontic treatment herein; after this period, the bracket needs to possess lower bond strength to ease removal without damaging the enamel structure [26].

Regarding fracture pattern analysis, the MDP-containing composite generated similar results to the positive control whereas the GDMA-P composite presented more adhesive failures due to its lower chemical interaction with the substrate [27]. Concerning MDP-based adhesives, the mode of application yields different bond strengths and MDP-Ca salt formation. Recently, it has been demonstrated that multi-layer application [28] and extended application time [29] provide improved adhesion, which might also be considered for the orthodontic bracket bond. The negative control group presented a large number of premature failures and almost absent enamel etching, thereby providing very low adhesion. Contrariwise, the positive control showed mixed/adhesive failures generating enamel fractures and remnants of composites that are more difficult to remove without damaging the tooth surface. The results of the present study are limited to applications with ceramic brackets, experimental composition used, and study design. Indeed, the outcomes may vary when using further types of orthodontic accessories and different concentrations of acidic monomers. However, the present MDP-containing self-adhesive orthodontic composite has demonstrated feasible capacity to be employed clinically. Indeed, its use may afford faster and less laborious bracket bonding as well as less enamel damage. Therefore, future projects should propose clinical trials to compare such a composite with traditional orthodontic bracket bonding techniques. 

## 5. Conclusions

The experimental orthodontic composite incorporated with MDP achieves shear bond strength performance similar to a conventional (three-step) composite and displays similar mechanical properties. However, the presence of an acidic functional monomer may reduce the degree of conversion of innovative self-adhesive orthodontic composites.

## Figures and Tables

**Table 1:** Spreading of groups for shear bond strength and clinical fracture pattern.

Groups		Procedure Description
Positive control immediate (POS-IM)	Positive control after thermocycling (POS-TC)	Enamel acid-etching + adhesive application + control composite insertion
Negative control immediate (NEG-IM)	Negative control after thermocycling (NEG-TC)	Control composite insertion
MDP immediate (MDP-IM)	MDP after thermocycling (MDP-TC)	Insertion of composite with MDP acidic monomer
GDMA-P immediate (GDMA-P-IM)	GDMA-P after thermocycling (GDMA-P-TC)	Insertion of composite with GDMA-P acidic monomer

**Table 2:** Outcomes of the degree of conversion (%).

Groups	Degree of Conversion (%)
Control composite	84.6 ± 4.3% ^a^
GDMA-P	58.9 ± 4.0% ^b^
MDP	23.0 ± 5.2% ^c^

Different lowercase letters depict statistical differences (*p* < 0.05).

**Table 3:** Outcomes of shear bond strength.

Group	Immediate	After Thermocycling	Fracture Pattern		
			Mixed	Cohesive	Adhesive
Positive	8.47 ± 0.88 MPa ^aA^	1.12 ± 0.52 Mpa ^aB^	7/12	3/12	2/12
GDMA-P	6.17 ± 1.46 Mpa ^bA^	1.34 ± 1.04 Mpa ^aB^	4/12	-	8/12
MDP	7.07 ± 2.69 Mpa ^abA^	1.45 ± 0.60 Mpa ^aB^	6/12	4/12	2/12
Negative	2.37 ± 1.81 Mpa ^cA^	0.11 ± 0.27 Mpa ^aB^	1/12	-	11/12

Different uppercase letters in each row and lowercase letters in each column depict statistical differences (*p* < 0.05).
